# What is a Freestanding Emergency Department? Definitions Differ Across Major United States Data Sources

**DOI:** 10.5811/westjem.2020.3.46001

**Published:** 2020-04-16

**Authors:** Darya M. Herscovici, Krislyn M. Boggs, Ashley F. Sullivan, Carlos A. Camargo

**Affiliations:** Massachusetts General Hospital, Department of Emergency Medicine, Boston, Massachusetts

## Abstract

**Introduction:**

Despite the growing number of freestanding emergency departments (FSED) in the United States (US), FSED definitions differ across major US data sources of healthcare facilities and use. We compare these sources and propose a universal definition of FSED (and its two major types) to improve communications regarding these facilities and their patients.

**Methods:**

We collected definitions of FSEDs from 11 national data sources using their websites, email, and telephone communications. For each source, we asked how they define FSEDs, whether being open 24/7 is a requirement to be called an ED, and whether they maintain a dataset of FSEDs.

**Results:**

Definitions varied across the data sources. All sources recognize FSEDs in their definitions, regardless of type; only one (the National Health Intervew Survey) does not differentiate them from other EDs. Five of the 11 sources (45%) omit autonomous FSEDs from their definitions and do not separately identify satellite FSEDs from their affiliated hospitals. One source does separately identify satellite FSEDs from their affiliated hospitals, but also omits autonomous FSEDs. Furthermore, three of the 11 sources (27%) do not require being open 24/7, while all others (73%) employ this criterion. Six of the 11 (55%) maintain datasets of FSEDs using their definition.

**Conclusion:**

As FSEDs continue to change the landscape of emergency care, it is important that they also be represented in national ED data sources. The current differences in the definition of an FSED make it difficult to provide accurate and longitudinal analysis for these facilities and patients who receive services at these facilities. We propose a universal definition of FSEDs as described by both the American College of Emergency Physicians and the National Emergency Department Inventory. Implementing a standard definition would facilitate a more accurate representation of FSEDs in national data sources and enhance ongoing efforts to improve the quality of emergency care delivered in FSEDs.

## INTRODUCTION

The American College of Emergency Physicians (ACEP) defines a freestanding emergency department (FSED) as an emergency facility that is not physically connected to inpatient services.^1^ In recent years, the number of FSEDs in the United States (US) has grown exponentially.^2^ According to the National Emergency Department Inventory (NEDI)-USA, a database containing all 24/7/365 non-federal EDs, in 2001 1% (50/4,884) of all US EDs were FSEDs.^3^ In 2016, however, the Medicare Payment Advisory Commission (MedPAC) reported that FSEDs accounted for 11% (566/5,200) of all EDs nationwide.^4^

This increase in FSEDs is in part a result of a 2004 Medicare reimbursement policy change that allowed payment for services provided in FSEDs.^4^ However, this policy only applies to hospital-affiliated (satellite) FSEDs and does not include non-hospital-affiliated (autonomous) FSEDs. The introduction of this policy presented an important difference between the two major types of FSEDs. Differences between these two types are not always indicated in major US data sources, which can lead to inaccurate representation of the ED landscape.

To characterize the magnitude of this data gap, we compared how US data sources define FSEDs and propose a universal definition of these facilities that allows for clear distinctions between two major FSED types. Adoption of this terminology would facilitate a more accurate representation of FSEDs in national data sources, communication about FSEDs, and efforts to improve the quality of emergency care delivered in FSEDs.

## METHODS

We compiled information from the seven sources presented in the 2010 profile of national ED sources by Owens et al.^5^ Data from the Hospital Market Profiling Solution, originally supported by Service Management Group, has since been integrated into the IQVIA OneKey Hospital Database following ownership transitions. Additional FSED definitions from four sources – ACEP; the Drug Abuse Warning Network (DAWN); the Emergency Department Benchmarking Alliance (EDBA); and the Centers for Medicare & Medicaid Services (CMS) – were obtained from a May 2019 online search using the criteria “national emergency department databases.”

We collected information from each source using website, email, and telephone communication. When we collected information from only one representative from an organization, we contacted these organizations a second time six months later to confirm the definition. For each source, we determined whether a facility must be open 24/7 to be classified as an FSED and whether the source maintains an ED dataset. We also determined whether they separately identify each type of FSED, and we extracted unique terminology used to identify the major types of FSED (eg, satellite vs autonomous). We employed the term satellite FSED to encompass all hospital-affiliated FSEDs, and autonomous FSED to encompass all non-hospital-affiliated FSEDs as outlined by Sullivan et al.^2^ We investigated the number of FSEDs of each type and their visit volumes in 2017 using data from NEDI-USA.^6^ Data are presented as proportions and medians with interquartile ranges (IQR) from Stata version 14.1 (StataCorp. College Station, TX).

### Data Sources

ACEP is a professional organization of US-based emergency physicians with >38,000 members. We obtained the organization’s FSED definition from the September 2019 Policy Compendium on its website.^1^ The American Hospital Association Annual Survey Database uses data from its annual survey that asks hospitals about facility characteristics.^7^ We obtained its FSED definition in a conversation with Vice President of Policy Research, Analytics, and Strategy A. Weslowski and Director of Health Analytics and Policy C. Vaz (March 2018).

CMS maintains multiple datasets, including a Provider of Services file where data on characteristics of healthcare facilities are kept.^8^ We obtained their FSED definition using the June 2018 MedPAC Report. DAWN is a nationwide public health surveillance system that tracks drug-related ED visits in a representative sample of US EDs.^10^ We obtained its FSED definition on the DAWN website under its glossary of terms. EDBA maintains a database of performance metrics from over 2,500 hospitals.^11^ We obtained its FSED definition in a conversation with Vice President J. Augustine (January 2020).

IQVIA maintains a dataset of hospitals and outpatient centers.^12^ We obtained its FSED definition in a conversation with the sales solution specialist DE Franz (January 2020). NEDI-USA is maintained by the Emergency Medicine Network at Massachusetts General Hospital. It contains an inventory of all US EDs that includes facility characteristics.^3^ We obtained the FSED definition using the 2007 profile of FSEDs by Sullivan et al.^2^

The Nationwide Emergency Department Sample (NEDS) is sponsored by the Agency for Healthcare Research and Quality. It includes data on hospital and patient characteristics for a representative sample of EDs.^13^ We obtained its FSED definition using the 2017 NEDS Introduction and a conversation with lead technical advisor M. Barrett (January 2020).^14^ The National Electronic Injury Surveillance System is run by the National Center for Injury Prevention and tracks injury-related ED visits in a representative sample of hospitals within the US.^15^ We obtained its FSED definitions during a telephone conversation with the director T Schroeder (February 2020).

The National Hospital Ambulatory Medical Care Survey (NHAMCS) is an annual survey conducted by the National Center for Health Statistics of the US Centers for Disease Control and Prevention. This survey collects information on hospital departments and ambulatory surgery centers.^16^ The FSED definition was obtained using the 2017 NHAMCS micro-data file documentation.^17^ The National Health Interview Survey is a household-based survey of the US population that collects information on healthcare utilization, status, and coverage for members of the selected household.^18^ FSED definitions were obtained through a telephone call with statistician M. Martinez (January 2020).

## RESULTS

**Figure 1:**
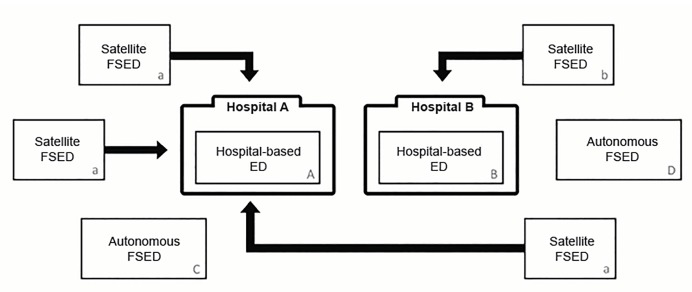
Example of the relation between hospital-based emergency departments, satellite freestanding emergency departments, and autonomous freestanding emergency departments. This schematic contains eight total EDs: two hospital-based EDs (located within “Hospital A” and “Hospital B”), and six FSEDs. The three satellite FSEDs “a” are affiliated with “Hospital A”, and satellite FSED “b” is affiliated with “Hospital B.” The two autonomous FSEDs, “Autonomous C” and “Autonomous D,” operate independently of any hospital affiliation. *ED*, emergency department; *FSED*, freestanding emergency department

Overall, FSED definitions vary across the 11 major US data sources (Table 1). Among the 11 sources, all recognize FSEDs within their definition. Three (27%) do not require that FSEDs are open 24/7. Five sources (45%) omit autonomous FSEDs and do not separately identify satellite FSEDs as part of their FSED definitions. Furthermore, multiple terms are used across different datasets when describing FSEDs. Across the 11 sources, four different names are used to describe satellite FSEDs and two different names are used to describe autonomous FSEDs. Figure 1 shows the relation between satellite and autonomous FSEDs, with respect to hospital-based EDs. Upon comparison, only NEDI-USA maintains an ED dataset that requires FSEDs to be open 24/7, separates satellite and autonomous FSEDs, and maintains annual ED visit volumes for these centers.

### FSED Types and Visit Volume

Since NEDI-USA is the only data source that separates FSEDs by type and includes facility-specific visit volumes, we present results from this dataset. In 2017, NEDI-USA reported that out of 5,455 EDs, 669 (12%) are FSEDs. Among FSEDs, 408 (61%) are satellite FSEDs and 261 (39%) are autonomous FSEDs. The three states with the most FSEDs are Texas (373 FSEDs), Ohio (45), and Colorado (44). In Texas, 246 (66%) of FSEDs are autonomous, while 127 (34%) are satellites. Most FSEDs in Ohio and Colorado are satellites (98% and 77%, respectively). Of 159,531,391 ED visits nationwide in 2017, 7,387,966 (5%) were from satellite FSEDs while 1,587,371 (1%) were from autonomous FSEDs. The median visit volume for satellite FSEDs in 2017 was 17,250 (IQR = 9,348–21,900), while the median visit volume for autonomous FSEDs was 4,530 (IQR = 2,920–8,433).

## DISCUSSION

We found important definitional differences for FSEDs among 11 major US data sources. Specifically, datasets differ in whether or not they separate hospital-affiliated (satellite) FSEDs from non-hospital-affiliated (autonomous) FSEDs. These distinctions complicate efforts to obtain accurate and representative national ED data and suggest that one should use caution when interpreting FSED data, depending on the source. Accurate information about FSEDs is essential to both research efforts and legislation related to this rapidly growing part of the emergency care landscape. There is also statewide variation in policies regarding FSEDs. A 2016 study determined that 29 states have no regulations about FSEDs (either to encourage or limit them).^19^ There are only four states where autonomous FSEDs are legal. The diversity of state FSED policies is a reflection of the diversity of FSED definitions.

In 2014, ACEP published essential criteria for FSED facilities.^1^ These requirements included 24/7 availability (as is typically required for any ED) and distinctions for “hospital-affiliated” and “non-hospital-affiliated” FSEDs. Despite the publication of these criteria, the ACEP definition has not yet been adopted by several national organizations (Table 1). Most often, FSEDs are not identified separately within datasets. Rather, they are imbedded within the hospitals they are affiliated with, or are excluded from their sampling frame.

Based on our analysis, NEDI-USA is the only source that maintains an up-to-date inventory that abides by the guidelines published by ACEP and has been doing so since the mid-2000s.^3^ Additionally, the NEDI-USA FSED definition allows for all FSED locations to be included within datasets and for differentiation among satellite vs autonomous FSEDs. Without this differentiation, essential ED information is lost, and datasets may no longer accurately reflect the facilities that deliver emergency care.

## LIMITATIONS

Despite identifying 11 major US data sources, there may be additional sources that classify FSEDs. We are not aware of such sources, nor are the multiple individuals we contacted at the identified organizations. Regardless, among the identified datasets, there exist major differences that merit discussion and support adoption of standard terms (ie, ACEP/NEDI-USA definition) to improve clarity. Additionally, the amount and nature of information accessible for each organization and their corresponding dataset varied. However, whenever information was not clearly available online, we collected additional data from the organization by phone or email and collected the names and positions of the individuals we spoke to.

Lastly, because of the heterogeneity of the 11 sources, direct comparisons were not possible. Instead, we compared sources that keep inventories versus sources that conduct patient interviews versus sources that only maintain FSED definitions. However, we were able to identify comparable criteria despite the fundamental differences in sources and we demonstrated important differences.

## CONCLUSION

Currently, there are discrepant definitions for FSEDs among major US data sources. Universally employing the ACEP/NEDI-USA definition would allow FSEDs to be individually identified and listed in national ED datasets. This would allow for future research to more accurately characterize all of US ED care and facilitate ongoing efforts to improve the quality of emergency care, including that provided in the growing number of FSEDs.

## Figures and Tables

**Table 1:** Comparison of freestanding emergency department definitions among major US data sources.

Type of ED source	24/7 operation requirement	Maintains ED dataset	Separately identifies autonomous FSEDs (ie, unaffiliated with hospital)	Separately identifies satellite FSEDs (ie. affiliated with hospital)	Unique terminology	Notes
American College of Emergency Physicians (ACEP)^1^	Yes	No	Yes	Yes	Hospital Outpatient Department (HOPD): Satellite FSED Independent Freestanding Emergency Center (IEFC): Autonomous FSED	
American Hospital Association (AHA) Annual Survey Database^7^	Yes	Yes	No	No	Satellite Off Campus ED (OCED): Satellite FSED	Groups information from satellite FSEDs with its affiliated parent hospital
The Centers for Medicare & Medicaid Services (CMS)^8^	No	Yes	No	No		FSED needs to be owned and operated by a Medicare participating hospital, or meet requirements to seek participation in Medicare as a “hospital specializing in emergency services.” These facilities do not have a 24/7 requirement.
Drug Abuse Warning Network (DAWN)^10^	Yes	No	No	No	OCED: Satellite FSED	Uses AHA criteria
Emergency Department Benchmarking Alliance (EDBA)^11^	Yes	Yes	No	Yes		
IQVIA OneKey Hospital Database^12^	No	Yes	Yes	Yes		“FSED” includes any outpatient center with a site specialty of Emergency Medicine
National Emergency Department Inventory (NEDI)^3^	Yes	Yes	Yes	Yes		
Nationwide Emergency Department Sample (NEDS)^13^	Yes	No	No	No		Uses AHA criteria
National Electronic Injury Surveillance System (NEISS)^15^	Yes	No	No	No	HOPD: Satellite FSED	Uses AHA criteria
National Hospital Ambulatory Medical Care Survey (NHAMCS)^16^	Yes	No	No	No		Only considers FSEDs as EDs unaffiliated with a hospital
National Health Interview Survey (NHIS)^18^	No	No	Yes	Yes		No differentiation between FSEDs and hospital-based EDs, all classified under ED

*ED*, emergency department; *FSED*, freestanding emergency department; *US*, United States.
